# Misplaced Urinary Catheter in the Ectopic Ureter in a Female With Previously Undiagnosed Duplex Ureter: A Rare Case

**DOI:** 10.7759/cureus.31139

**Published:** 2022-11-05

**Authors:** Mahsa Danaie, Ahmadreza Nezameslami, Mohammad Mishan, Bahareh Mehdikhani, Zeinab Mansouri

**Affiliations:** 1 Department of Obstetrics and Gynecology, Abadan University of Medical Sciences, Abadan, IRN; 2 Department of Orthopedic Surgery, Tehran University of Medical Sciences, Tehran, IRN; 3 Department of General Surgery, Abadan University of Medical Sciences, Abadan, IRN; 4 Department of Radiology, Iran University of Medical Sciences, Tehran, IRN; 5 Department of Obstetrics and Gynecology, Imam Khomeini Hospital, Tehran University of Medical Sciences, Tehran, IRN

**Keywords:** ectopic ureter, complication, duplex kidney, ureteric catheterization, misplaced foley catheter

## Abstract

Unintentional Foley catheter placement into a ureter is an extremely rare and life-threatening complication that can be easily prevented by early diagnosis. A misplaced Foley catheter should be suspected in the presence of abdominal pain and oliguria after bladder catheterization, and congenital variation of the urinary tract can complicate this procedure. We reported the third case of misplaced insertion of a Foley catheter into the upper moiety ureter of a duplex kidney in a 19-year-old post-partum woman. The patient complained of the right flank and pelvic pain and oliguria one day after urinary catheter insertion. Duplicated right kidney, upper pole moiety mild hydronephrosis and malpositioned Foley catheter balloon in the right ectopic ureter were reported on ultrasonography which was confirmed by a CT scan. The catheter was removed easily without any complications.

## Introduction

The duplex kidney is one of the common urinary tract anomalies, which is reported in 0.8% of the autopsy series [1,2]. Women are affected twice as much as men, and most of them are asymptomatic [1,3]. Bladder catheterization is a very common and routine urological procedure [4]. It can be associated with complications, the most common of which are urinary tract infections and traumatic injuries [5].

Unintentional Foley catheter placement into a ureter is an extremely rare complication, which Most of them have been documented in females and patients with a neurogenic bladder [6]. Manifestations may include persistent leakage of urine around the urethral catheter, pain, blocked catheter and sometimes, it may be asymptomatic. Obstructive hydronephrosis, injury and rupture of the ureter could happen [7,8]. This entity could be potentially life-threatening but may be prevented by early diagnosis.

Only 26 cases of ureteric catheterization are reported within the literature as late as 2022 [9]. More rarely, only two cases of them are described in the duplicated pelvicalyceal system [10,11]. To our knowledge, this is the third case to be reported.

## Case presentation

The patient was a 19-year-old post-partum woman who had a history of term vaginal delivery five weeks before presentation. Her relatives complained of mood problems, severe anorexia and reduced bonding with her baby. After Psychiatric consultation, she was admitted for further evaluation in terms of psychosis and postpartum depression. In the past medical and drug history of the patient, there was no noteworthy point except contrast media allergy.

**Figure 1:**
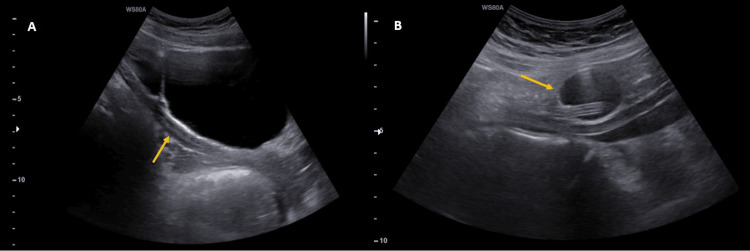
Ultrasonography findings Foley catheter in the ectopic ureter draining to the urethra (A) misplaced balloon of Foley catheter in the right lower quadrant near iliac arteries (B)

On the first day of admission, 16 French Foley catheter was placed in her bladder with inflation of the 10-cc balloon by paramedical staff due to unsteady gait. On the second day, she complained of the right flank and pelvic pain. There was a significant reduction in urinary output as she was only able to make 150cc over 12 hours. The laboratory work-up revealed blood urea nitrogen (BUN) of 25 mg/dL, serum creatinine of 1.3 mg/dL and 3+ red blood cells (RBC) in urinalysis. Duplicated right kidney and upper pole moiety mild hydronephrosis was reported at ultrasonography. A malpositioned Foley catheter balloon at the right lower quadrant in the right ectopic ureter was found (Figures 1A, 1B).

**Figure 2:**
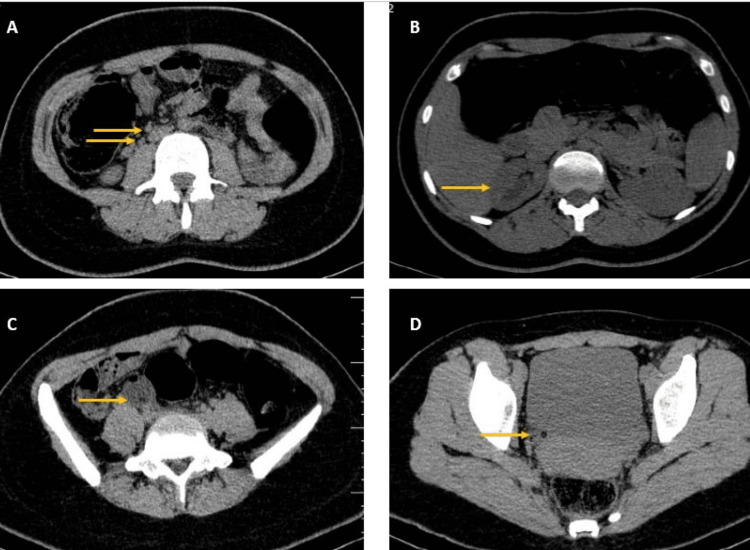
CT scan without contrast findings Duplex ureters (A), mild hydronephrosis of upper moiety (B), Misplaced balloon of Foley catheter in the right lower quadrant (C), and Foley catheter in the ectopic ureter draining to urethra outside of the bladder (D). CT: Computed Tomography

**Figure 3:**
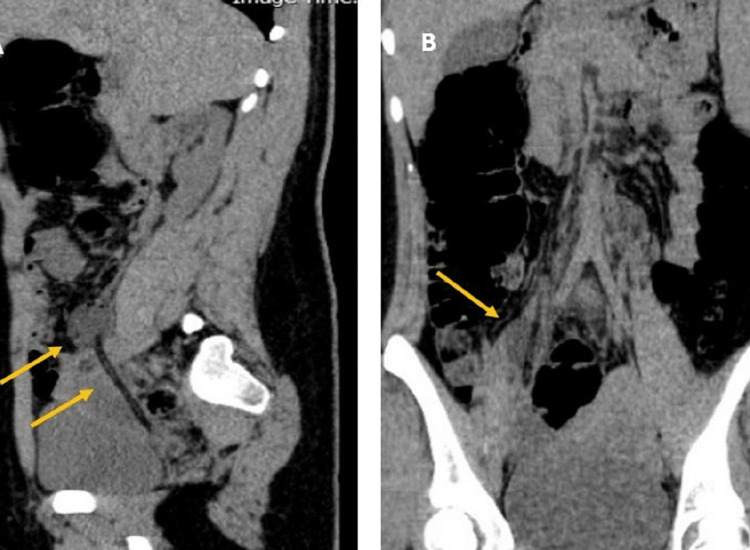
CT scan without contrast findings Tip of urinary catheter in the middle part of the ectopic ureter on the right side in sagittal (A) and coronal (B) planes CT: Computed Tomography

Because of the previous history of contrast media allergy, a computed tomography (CT) scan without contrast was taken, inevitably, which confirmed the ultrasound findings. As far as can be seen in the CT scan without contrast, the right kidney was duplicated, and the ureter of the upper moiety was ectopically drained into the proximal of the urethra. A misplaced Foley tube balloon was seen in the middle part of the ectopic ureter on the right side leading to mild hydronephrosis of the upper pole right kidney (Figures 2A-2D, 3A, 3B).

**Figure 4:**
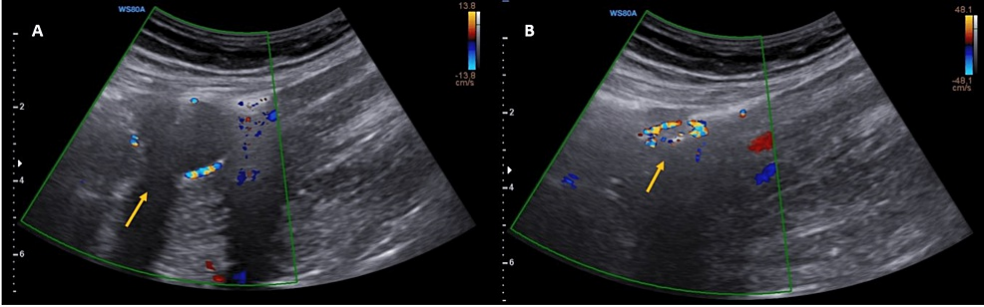
Ultrasonography of misplaced Foley catheter balloon Before (A) and after (B) deflation

After the urology consultation, an attempt was made to remove the Foley catheter under ultrasound guidance (Figures 4A, 4B). The balloon easily deflated which was visible in the ultrasound and the Foley catheter was removed gently with no incident. There was not any detectable urine jet from the more proximal ureter on concurrent ultrasound. In a short time after the procedure, the oliguria resolved.

The patient was observed for 48 hours to ascertain the resolution of abdominal pain and improved urinary output. During the time of observation, daily evaluation with ultrasound was conducted to exclude abdominal fluid collection and monitor the gradual resolution of hydronephrosis. Afterward, she was referred to a tertiary care center for psychiatric management and due to possible stricture, that may arise from inadvertent inflation of the balloon in the ureter, long-term follow-up imaging for hydronephrosis was recommended.

## Discussion

Bladder catheterization is a very common urological procedure [4]. It can be associated with complications including urinary tract infection, trauma, inflammatory reactions, urethral stricture and false route, which could be presented with pain, bleeding, catheter blockage and expulsion [4,5].

Unintentional Foley catheter placement into a ureter is an extremely rare complication. Manifestations may include persistent leakage of urine around the urethral catheter, pain, blocked catheter, partial or complete ureteric rupture, obstructive hydronephrosis and sepsis or could be an incidental diagnosis [7,8]. Our patient presented with flank pain and oliguria.

Female patients are three times more at risk than males due to having a much shorter urethra. The majority of patients are under long-term catheterization, mostly due to neurogenic bladder with resultant impaired sensation. Inadvertent fixation of the catheter into the ureter can occur during the first insertion of the catheter in patients without sensory problems. This group like our patient is more likely to complain of lower abdominal or flank pain, which will prompt early diagnosis [6,8].

Other predisposing factors are contracted bladders, physiologic changes in pregnancy, congenital anatomic abnormalities like ectopic ureters, patulous ureters and iatrogenic factors. Small caliber urethral catheters and catheterization in the empty bladder may increase the risk of misplacement, too [7,8,12-14].

Ultrasound examination can be performed as a first choice to detect the location of the suspected malpositioned catheter. On ultrasound, a Foley balloon may be detected in the abdomen along the course of the ureter as a spherical hypoechoic or anechoic structure. Proximal hydronephrosis could be evident due to ureteral obstruction. In the case of ureteral rupture, urine jets may be detectable using a grey scale and color Doppler ultrasound [13]. As mentioned before, in the presented case, the misplaced location of the Foley catheter balloon in the ectopic ureter was well demonstrated in the ultrasound examination.

A Foley balloon as a spherical structure with water attenuation and Hydroureteronephrosis is easily recognizable in a CT scan without contrast. However, a contrast-enhanced CT scan is the preferred imaging method to evaluate complications particularly ureteral rupture, contrast leakage to the abdominopelvic cavity, and the need for subsequent interventional measures [8,13]. We performed a non-contrast CT scan due to the patient's history of contrast media allergy. As well as our case, if the ureter is not damaged, conservative treatment is enough by removing or replacing the Foley catheter.

The most likely long-term complication of a misplaced ureteral Foley catheter is ureteral stricture especially when the balloon is inflated. Subsequently, upper urinary tract obstruction could develop. Imaging modalities such as ultrasound or CT scans are necessary for monitoring [8]. Since we did not have the possibility of long-term observation due to the patient’s psychiatric problems, we just recommended a follow-up ultrasound to check for hydronephrosis.

Luo et al. reviewed 20 cases of ureteral insertion of the catheter, seven patients developed ureteric rupture; seven patients developed pyelonephritis; and seven patients had no adverse outcomes. The aberrant catheter was removed or changed easily in more than half of them without any further intervention, as in our patient [8].

Ureteric catheterization could be avoidable by the following tips: the length of the catheter inside the bladder should not be more than 6-8 cm, the balloon should be inflated only after urine is seen to be draining, bedside ultrasonography is suggested in high-risk individuals after catheter placement to confirm that the tip of the catheter is in the bladder and by paying attention to the patient's symptoms, including pain and decreased urine output after the procedure [6,8,15].

Only 26 cases of ureteric catheterization were reported within the literature as late as 2022 [9]. More rarely, only two cases of them were described in the duplicated pelvicalyceal system. One of them was a 68-year-old woman who had undergone laparotomy for de-bulking surgery of malignancy. The unintentional insertion of the catheter into the previously undiagnosed duplex ureter leads to false intraoperative identification of the female anatomy and injury to the ureter. The ureter was repaired surgically [10]. Another case was a 28-year-old full-term pregnant woman, urinary catheter was fixed which shown inserted into the upper moiety ureter of a duplex kidney. The catheter was removed with ureteroscopic forceps [11]. To our knowledge, this case is the third to be reported.

## Conclusions

A misplaced urinary catheter into the ureter is a very uncommon entity that late diagnosis can lead to serious complications. In this case, we show how congenital anomalies can complicate routine bladder catheterization. The patient's symptoms should be paid attention to after catheter placement. In case of suspicious symptoms such as pain and decreased urinary output; available modalities like ultrasound could be used to check the location of the catheter. In this study, the role of ultrasound in the timely diagnosis of the misplaced catheter was well shown in a patient with contrast media allergy. In addition to a CT scan, an ultrasound can also identify the exact location of the misplaced catheter and even the complications if it is performed by expert radiologists.
